# Antibiogram and molecular characterization of extended-spectrum β-lactamase-producing pathogens implicated in chronic suppurative otitis media

**DOI:** 10.11604/pamj.2023.46.108.41156

**Published:** 2023-12-19

**Authors:** Francis Amadi Ibiam, Eze Egwu, Ikechukwu Benjamin Moses, Chidinma Stacy Iroha, Amali Adekwu, Godwin Obasikene, Monday Agbonifo, Ifeanyichukwu Romanus Iroha

**Affiliations:** 1 Department of Otorhinolaryngology, Alex Ekwueme Federal University, Ndufu Alike Ikwo, Nigeria; 2 Department of Medical Laboratory Science, Alex Ekwueme Federal University Teaching Hospital, Abakaliki, Nigeria,; 3 Department of Applied Microbiology, Faculty of Science, Ebonyi State University, Abakaliki, Nigeria,; 4 Department of Pharmacy, Alex Ekwueme Federal University Teaching Hospital, Abakaliki, Nigeria,; 5 Department of Otorhinolaryngology, Federal University of Science Otukpo, Otukpo, Nigeria,; 6 Department of Otorhinolaryngology, Nnamdi Azikiwe University, Nnewi, Nigeria,; 7 Department of Otorhinolaryngology, Ambrose Ali University, Ekpoma, Nigeria

**Keywords:** Chronic suppurative otitis media, extended-spectrumβ-lactamase, gram-negative bacteria, antimicrobial resistance, ear discharge

## Abstract

**Introduction:**

beta-lactamase-producing bacteria, especially extended-spectrum beta-lactamase (ESBL) producers have strong clinical relevance and have been implicated in chronic suppurative otitis media (CSOM) treatment failures. This study aimed to determine the frequency, antibiogram, and molecular characteristics of ESBL-producing gram-negative bacterial (GNB) pathogens isolated from patients with CSOM.

**Methods:**

three hundred (300) ear swab samples collected from patients with active CSOM were analysed using standard microbiological techniques. Antibiogram of pathogens was determined by Kirby-Bauer disk diffusion technique. Phenotypic detection and molecular characterization of ESBL-producing GNB pathogens were performed by double disk synergy test (DDST) and polymerase chain reaction (PCR).

**Results:**

Escherichia coli and P. aeruginosa were more prevalent among CSOM patients with a duration of discharge >2 weeks. The frequency of ESBL producers among the GNB pathogens was 18.3%. Isolates were generally multidrug-resistant but very susceptible (100% - 70.4%) to ciprofloxacin, imipenem, and amikacin. Multiple antibiotic resistance values of the isolates ranged from 0.7-0.8. Polymerase chain reaction showed that blaSHV (47.6%) was the most predominant ESBL genotype. This was followed by blaTEM (25.2%) and blaCTX-M (10.7%) as the least predominant ESBL gene. Concomitant expression of ESBL gene was observed in 13.6% of the isolates.

**Conclusion:**

this study reported the occurrence and spread of β-lactamase-producing bacteria in patients with CSOM infections. It is therefore very crucial to screen for antibiotic-resistant pathogens at early stages of CSOM infections, for proper antimicrobial therapy and to curb the increasing spread of antimicrobial resistance.

## Introduction

Middle ear inflammation with a perforated tympanic membrane, accompanied with ear discharges persisting for about 14 days is referred to as Chronic suppurative otitis media (CSOM) [1,2]. Chronic suppurative otitis media is distributed worldwide and ranks the second cause of hearing loss, especially among immunocompromised patients and children [3]. Due to a lack of education, poor nutrition, and poor hygienic practices in the developing world, the incidence of CSOM has increased [4]. The global disease burden of CSOM accounts for about 28,000 deaths with over 2 million hearing disabilities due to the irreversible destruction of the middle ear by bacterial pathogens [4]. Increasing trends of antimicrobial resistance rates by gram-negative bacteria (GNB) to ear infections worldwide are continually reported. The synthesis of beta-lactamases, especially ESBLs is one of the most significant resistance mechanisms by GNB reported worldwide [5]. Extended-spectrum beta-lactamases (ESBL) are usually plasmid-encoded enzymes with the ability to inactivate or hydrolyze cephalosporins (first, second, and third generations), penicillin, and even monobactams [2]. The genes (such as CTX-M, SHV, TEM, VEB, GES, and PER) encoding these ESBL enzymes are frequently detected in *Klebsiella* and *E.coli* species; and are transferable via the plasmid through horizontal gene transfer from one bacterium to another [5,6]. Considering the global threat of antimicrobial resistance in healthcare settings such as increased length of hospital stay, increased healthcare costs, and higher mortality rate [6]; studies on the frequency, antimicrobial susceptibility profiles, and molecular characteristics of ESBL-producing GNB pathogens implicated in ear infections are still scarce, and at the same time, indispensable. Also, baseline information depicting the magnitude of beta-lactamase-producing bacteria implicated in CSOM cases is limited and attests to reported poor treatment outcomes in patients. Previous studies on the etiologies of CSOM did not properly address its burden, especially by β-lactamase-producing GNB pathogens in ear infections. Meanwhile, this study reports the spread of multidrug-resistant ESBL-producing Gram-negative bacterial pathogens harboring ESBL genes in CSOM patients.

## Methods

**Area of study:** this study was done at the Federal University Teaching Hospital (AE-FUTHA), Abakaliki, Nigeria. Abakaliki is the capital city of Ebonyi State, South-eastern Nigeria. The hospital provides services such as surgical, medical, ear-nose-and-throat (ENT), and intensive care. The hospital has more than 1200 beds and provides healthcare services to thousands of people in the study regions and even referrals from nearby regions [7]. The major occupations of people+ in Abakaliki are farming and trading; there are also civil servants and students, and all these people engage in busy activities of life [8].

**Ethics:** ethical approval for this study was granted by the ethics and research committee of AE-FUTHA with reference number: SMOH/ERC/042/21. Every fundamental study was done in accordance with the ARRIVE guidelines and the World Medical Association (WMA) Declaration of Helsinki [9].

**Sampling:** random sampling was used in selecting CSOM patients. Recruitment of subjects into the study was done as they visited the hospital until the required number was obtained with strict implementation of both the inclusion and exclusion criteria.

**Inclusion criteria:** all study participants with active CSOM i.e, draining ears resulting from CSOM of two weeks and above and perforated tympanic membranes with active purulent discharge were included.

**Exclusion criteria:** patients with discharges < 14 days duration, discharges with intact tympanic membrane (otitis externa), and those undergoing antimicrobial therapy (topical or systemic) within 7 days before data collection were excluded.

**Clinical demographic and medical history:** using a structured questionnaire by the attending ENT specialist, vital information such as duration of discharge, sex, age, and unilateral or bilateral draining ears were obtained from study participants after medical examination.

**Sample collection:** three hundred (300) middle-ear discharges were collected under strict aseptic conditions by an ENT specialist with single mini-tip swabs after cleaning the external ear auditory canal with a spirit swab, as previously described by Molla *et al*. [10]. Bilateral draining pus specimens were collected from both ears of patients and swabs were immediately transported to the microbiology laboratory for analysis.

**Microbiological analysis:** swabs were firstly enriched in nutrient broth and were aerobically incubated overnight for 24 hours at 37°C. After overnight incubation, loopfuls of the turbid broth culture were streaked aseptically on sterile cetrimide agar, Eosin Methylene Blue agar, and MacConkey agar (Oxoid, UK), and thereafter incubated for 24 hours at 37°C. Identification of isolates was done morphologically concerning color and shape on the selective and differential media. Each atypical colony was purified on nutrient agar through successive streaking before physiological and biochemical identification such as gram staining, triple sugar iron agar, catalase, citrate utilization, oxidase test, indole production, carbohydrate utilization, and motility tests. Isolates were further identified using specific 16S rRNA primers by PCR [11].

**Extended-spectrum beta-lactamase production confirmation by double-disk synergy test:** this was done as previously described [12,13]. Overnight suspension of the 2^nd^ and 3^rd^ generation cephalosporin-resistant bacteria was standardized (0.5 McFarland turbidity standard) and swabbed aseptically on Mueller-Hinton agar (MHA). Amoxicillin-clavulanic acid [AMC (20/10μg)] was placed at the center of the inoculated MHA plate. Ceftazidime (30μg) and cefotaxime (30μg) discs were then placed at a distance of 15mm from the central disc (AMC-20/10μg) and thereafter incubated at 37°C for 18-24h. After the incubation period, a ≥ 5 mm increase or expansion in inhibition zone diameter (IZD) for either of the cephalosporins (ceftazidime and cefotaxime) tested in combination with amoxicillin-clavulanic acid compared with its Isothiazolidinone (IZD) when tested alone confirms ESBL production [12,13].

**Antimicrobial susceptibility studies:** the antibiogram of the CSOM isolates was done by disc diffusion technique according to the guidelines of the Clinical and Laboratory Standards Institute (CLSI) [12]. The following antimicrobials were tested against the isolates: Amikacin (10μg), amoxicillin (30μg), ceftazidime (30μg), amoxicillin/clavulanic acid (30μg), ceftriaxone (30μg), cefuroxime (30μg), cefotaxime (30μg), cefoxitin (30μg), ciprofloxacin (10μg), imipenem (30μg), ofloxacin (30μg), piperacillin (30μg), trimethoprim-sulfamethoxazole (25μg), and tetracycline (30μg). Results were interpreted as resistant or susceptible according to the CLSI guidelines [12].

**Multiple antibiotic resistance index (MARI):** this was determined using the description of Moses *et al*. [14]. The MAR indices was calculated as the number of antibiotics to which the GNB pathogens exhibit resistance: a) divided by the total number of antimicrobial agents tested against the GNB pathogens (b).

**Detection of ESBL-encoding Genes by PCR:** according to the manufacturer´s protocol, genomic DNA extraction of bacterial pathogens was performed using Zr Fungal/Bacterial DNA Miniprep™ kit (Zymo research, cat number: D6005). Polymerase chain reaction mix components comprised 12.5μL of Taq 2x Master Mix, 1μL each of 10μM forward and reverse primer, 2μL of DNA template, and then 8.5μL Nuclease water. Oligonucleotide primers used,6 including their amplicon sizes are shown in Table 1.

**Table 1:** oligonucleotide primers

Primers	Nucleotide sequence	Base pair (bp)
TEM-F	GCG GAA CCC CTA TTTG	1350
TEM-R	TCT AAA GTA TAT ATG AGT AAA CTT GGT CTG AC	
SHV-F	TTC GCC TGT GTA TTA TCT CCCTG	700
SHV-R	TTA GCG TTG CCA GTG YTC G	
CTX-M F	ATG TGC AGY ACC AGT AAR GTKATGGC	550
CTX-M R	TGG GTR AAR TAR GTSACC AGA AYC AGC GG	

## Results

Chronic suppurative otitis media patients infected with *E. coli* was higher among the age group of 21-30 years [13(4.3%)] while *K. pneumoniae* was predominant in patients aged 41 years and above with a frequency of 29 (9.7%), followed by the most predominant frequency of 42 (13.7%) for *P. aeruginosa coli, Klebsiella pneumoniae*, and *Pseudomonas aeruginosa* respectively (Table 2). *Escherichia coli* (9.0%) and *P. aeruginosa* (20.3%) were more prevalent among patients with duration of discharge >2 weeks, while *K. pneumoniae* was observed in 17.0% cases of discharge within the period of 2 weeks (Table 2). Gram-negative bacteria were more predominant in male patients with frequencies of 26 (8.7%), 40 (13.3%), and 63 (21.0%) for *Escherichia. coli, K. pneumoniae*, and *P. aeruginosa* respectively (Table 3). In the three hundred samples collected, *P. aeruginosa* accounted for 36.3% as the most prevalent bacterial pathogen recovered from CSOM patients; followed by *K. pneumoniae* and *E. coli* with frequencies of 22.3% and 13.7% respectively (Table 3). *Escherichia coli* was the most predominant ESBL producer with frequency of 9.0%; followed by *K. pneumoniae* (6.0%) and *P. aeruginosa* (3.3%) (Table 3). The ESBL-producing GNB pathogens (*K. pneumoniae, E. coli*, and *P. aeruginosa*) obtained from CSOM patients were generally resistant (60% -100%) to amoxicillin, amoxicillin-clavulanic acid, cefotaxime, cefuroxime, cefoxitin, ceftriaxone, ceftazidime, piperacillin, trimethoprim-sulfamethoxazole, and tetracycline but completely susceptible (100%) to amikacin and imipenem (Table 4). The average MAR index values of the ESBL-producing *Escherichia coli, Klebsiella pneumoniae*, and *Pseudomonas aeruginosa* were 0.7, 0.8, and 0.7 respectively (Table 4). Among the 55 ESBL-producing Gram-negative bacterial pathogens isolated, the most predominant ESBL gene was *bla*SHV (89.1%); followed by by *bla*TEM (47.3%), and *bla*CTX-M (20%) being the least predominant ESBL gene (Table 5). The proportion of *Escherichia coli, Klebsiella pneumoniae*, and *Pseudomonas aeruginosa* harbouring *bla* SHV were 45.5%, 29.1%, and 14.6% respectively (Table 5). *Bla* TEM gene was detected in *Escherichia coli* (27.3%), *Klebsiella pneumoniae* (12.7%), and *Pseudomonas aeruginosa* (7.3%). The frequency of *bla*CTX-M in *Escherichia coli, Klebsiella, pneumoniae*, and *Pseudomonas aeruginosa* were observed to be 12.7%, 5.5%, and 1.82% respectively. Concomitant expression of ESBL gene was observed in 25.5% of the isolates comprisingConcomitant expression of ESBL gene was observed in 25.5% of the isolates comprising *E. coli* (12.7%), *K. pneumoniae* (5%), and *P. aeruginosa* (7.3%) (Table 5).

**Table 2:** frequency distribution of gram-negative bacterial pathogens causing CSOM regarding demographic and clinical characteristic of patients

Patient information	Sample distribution	chronic suppurative otitis media bacteria n (%)		
Age (years)		*E. coli*	*K. pneumoniae*	*P. aeruginosa*
1-10	51	9 (3.0)	15 (5)	42 (13.7)
11-20	79	6 (2.0)	7 (2.3)	19 (6.7)
21-30	91	13 (4.3)	16 (5.3)	15 (5.0)
31-40	45	5 (1.7)	0 (0.0)	8 (2.7)
41 years and above	34	8 (2.7)	29 (9.7)	2(8.3)
**Gender**				
Male	131	26 (8.7)	40(13.3)	63 (21.0)
Female	169	15 (5.0)	27(9.0)	46 (15.3)
**Discharging ear**				
Bilateral	215	23 (7.7)	28 (9.3)	78 (26.0)
Unilateral	85	18 (6.0)	39 (13.0)	31(10.3)
**Duration of discharge**				
2 weeks	227	14 (4.7)	51 (17.0)	48(16.0)
>2 weeks	73	27(9.0)	16 (5.3)	61(20.3)
**Total**	**300**	**41 (13.7)**	**67 (22.3)**	**109 (36.3)**

**Table 3:** bacterial distribution and frequency of extended-spectrum beta-lactamase genes-producing gram-negative pathogens isolated from chronic suppurative otitis media patients visiting AEFUTHA

chronic suppurative otitis media Bacteria (n=300)	Occurrence rate (%)	Extended-spectrum beta-lactamases-positive (%)
*Escherichia coli*	41 (13.7)	27 (9.0)
*Klebsiella pneumoniae*	67 (22.3)	18 (6.0)
*P. aeruginosa*	109 (36.3)	10 (3.3)
**Total**	**217 (72.3)**	**55 (18.3)**
**N: number of samples**		

**Table 4:** antibiotic susceptibility pattern of ESBL-producing gram-negative bacteria isolated from CSOM patients

	*E. coli*		*K. pneumoniae*		*P. aeruginosa*	
Antibiotic (µg)	Resistance n (%)	Susceptible n (%)	Resistance n (%)	Susceptible n (%)	Resistance n (%)	Susceptible n (%)
Amikacin	0(0.0)	27(100)	0(0.0)	18(100)	0(0.0)	10(100)
Amoxycillin-clavulanic acid	27(100)	0(0.0)	16(88.9)	2(11.1)	8(80)	2(20)
Amoxicillin	27(100)	0(0.0)	18(100)	0(0.0)	10(100)	0(0.0)
Cefotaxime	27(100)	0(0.0)	18(100)	0(0.0)	1(100)	0(0.0)
Cefuroxime	27(100)	0(0.0)	1(94.4)	1(5.6)	10(100)	0(0.0)
Cefoxitin	2(100)	0(0.0)	18(100)	0(0.0)	10(100)	0(0.0)
Ceftriaxone	21(77.8)	6(22.2)	17(94.4)	1(5.6)	9(90)	1(90)
Ceftazidime	20(74.1)	7(25.9)	15(83.3)	3(16.7)	10(100)	0(0.0)
Ciprofloxacin	8(29.6)	19(70.4)	0(0.0)	1(100)	(20)	8(80)
Imipenem	0(0.0)	2(100)	0(0.0)	18(100)	0(0.0)	10(100)
Ofloxacin	18 (66.7)	9(33.3)	9(50)	9(50)	6(60)	(40)
Piperacillin	27(100)	0(0.0)	18 (100)	0(0.0)	10 (100)	0(0.0)
Tetracycline	27 (100)	0(0.0)	18 (100)	0(0.0)	9(90)	1(10)
Trimethoprim-sulfamethoxazole	27 (100)	0(0.0)	18(100)	0(0.0)	10 (100)	0 (0.0)
**Average MARI**	**0.7**		**0.8**		**0.7**	

**Table 5:** frequency of extended-spectrum beta-lactamases genes in gram-negative bacterial pathogens isolated from chronic suppurative otitis media patients

Extended-spectrum beta-lactamases gene	*E. coli* (%)	*K. pneumonia* (%)	*P. aeruginosa* (%)	Frequency (%)
TEM	15(27.3)	7(12.7)	4(7.3)	26(47.3)
CTX-M	7(12.7)	3(5.5)	1(1.82)	11(20)
SHV	25(45.5)	16(29.1)	8(14.6)	49 (89.1)
**Association of β-lactam gene**				
TEM/SHV/CTX-M	7(12.7)	3 (2.9)	4(7.3)	14 (25.5)

## Discussion

In this study, we investigated the antimicrobial susceptibility profiles, occurrence frequency, and types of ESBL genes harbored by GNB pathogens isolated from CSOM patients in Abakaliki, South-eastern Nigeria. Our study reported the isolation of ESBL-producing *K. pneumoniae, E. coli*, and *P. aeruginosa* from ear discharges of CSOM patients with bilateral and unilateral hearing loss. The predominance of Gram-negative bacterial (GNB) pathogens from bilateral ear discharges of CSOM patients in this study agrees with previous reports [15,16]. As evidenced in this study, bacterial proliferations in CSOM patients were more predominant in age group of 1-10 years (30.9%) and the age group of 41 years and above. Similar frequencies of bacterial pathogens in CSOM patients within 1-10 years of age have been reported [15,17,18] with the greatest disease burden experienced between 6 months and 18 months of age. At least 60% of children have experienced an episode by one year of age, and 17% have suffered at least 3 episodes of acute otitis media (AOM) [17] which may progress to CSOM with long-term effects on auditory processing, physiological and cognitive development, early communication, educational process, and language development; if not treated [19]. However, children and infants within the age group of 1-10 years have been reported to have a higher predisposition to ear infections than adults, especially due to the anatomy of children´s eustachian tubes [20]. Gram-negative bacterial pathogens in CSOM patients were more frequent in males than females in our study. It has been earlier reported that males have higher risk factors than females in the development and acquisition of otitis media [18]. The reason for this is still largely unknown as most studies involved random selection of cases. Unorthodox treatment approaches by caregivers [such as the use of unconventional ear drops and concoctions (oil and honey) into the middle ear] and poor hygienic practices, especially in low middle-income countries with insufficient financial resources, including Nigeria, may cause the proliferation of opportunistic pathogens, thereby leading to eustachian tube blockage of affected patients. The isolation frequency of GNB pathogens in CSOM cases in our study is in agreement with previous studies done elsewhere [10,21,22].

The frequency of ESBL-producing GNB (18.3%) in our study correlates with other studies [1,2,4]. However, no ESBL-producing GNB in CSOM patients was recorded in a previous study in Nigeria [23]. Extended-spectrum beta-lactamase producing bacterial pathogens in this study exhibited a high frequency of resistance (59.1% -100%) to cephalosporins (ceftazidime, ceftriaxone, cefoxitin, cefuroxime, and cefotaxime) and tetracyclines. Our study is in tandem with previous studies [24,25]. All the bacterial isolates in our study showed low resistance to ciprofloxacin (0-29.6%) which agrees with other studies [2,26]. Meanwhile, the susceptibility of bacterial pathogens implicated in CSOM cases to ciprofloxacin has been reported in a previous study [15]. The ESBL-producing GNB pathogens in this study expressed multidrug-resistant (MDR) phenotype with MARI values ranging from 0.7 - 0.8. This is in tandem with another report [4]. Our findings showed the rising trend of MDR β-lactamase-producing GNB pathogens in CSOM patients. This might be attributed to the misuse of antibiotics and the lack of appropriate antimicrobial resistance diagnosis for effective treatment. Interestingly, all the β-lactamase-producing bacterial strains in our study were completely susceptible (100%) to amikacin and imipenem. The effectiveness of these antimicrobial agents against CSOM bacterial pathogens substantiates reports in many pieces of literature [4,10,16,22]. Polymerase chain reaction results showed that *bla*SHV (89.1%) was the most predominant ESBL gene; followed by *bla*TEM (47.3%), *bla*CTX-M (20%) being the least predominant ESBL gene among the GNB pathogens. Exactly 20% of the *E. coli* isolates in our study haboured the *bla*CTX-M. This is in tandem with the 26% CTX-M-harbouring *E. coli* isolates reported in another study [27], even though the *bla*CTX-M frequency in our study was lower. In another study, CTX-M, TEM, and SHV were identified in 90.8% (109/120), 40.0% (48/120), and 10.8% (13/120) of uropathogenic *E. coli* strains respectively [28]. Concomitant expression of ESBL gene was noticed in 25.5% of the isolates in our study which may be linked to their observed MDR traits. This has also been reported by Alqasim *et al*. [29].

## Conclusion

Our study reported the occurrence frequency and spread of multidrug-resistant ESBL-producing GNB pathogens (*Klebsiella pneumoniae, Escherichia coli*, and *Pseudomonas aeruginosa*) harbouring *bla*SHV, *bla*TEM, and *bla*CTX-M resistance genes in CSOM patients. Concomitant co-expression of ESBL genes was observed among the GNB pathogens. This study advocates for good personal hygiene among patients to curb the increasing menace of CSOM infection, especially in children and among immunocompromised individuals who are mostly affected. However, more studies are needed to comprehensively understand the etiology, pathogenesis, and clonal spread of bacterial pathogens implicated in CSOM cases to help in the effective management of CSOM.

### What is known about this topic

- *Bacterial pathogens have been implicated in the global chronic suppurative otitis media infections and deaths;*
- *Gram-negative bacterial pathogens are important aetiological agents;*
- *Increasing trends of antimicrobial resistance by gram-negative bacteria (GNB) to chronic suppurative otitis media infections are continually reported worldwide*.

### What this study adds

- *Extended-spectrum beta-lactamase-producing Escherichia. coli, P. aeruginosa, and K. Pneumoniae with multidrug-resistant traits were isolated from chronic suppurative otitis media patients;*
- *Isolated gram-negative bacterial pathogens harbour clinically relevant extended-spectrum beta-lactamase-encoding genes such as blaTEM, blaSHV, and blaCTX-M;*
- *Higher chronic suppurative otitis incidences were observed mostly among children*.
